# Societies

**Published:** 1877-06

**Authors:** 


### SOCIETIES.

We have often, through the pages of the Register asked for reports from all dental societies, in respect to change of officers, times and places of meetings, etc., etc., so that the list of societies could be kept accurate.

A few respond to these requests very promptly, but by far the larger number utterly fail, except to complain of the inaccuracies in that list.

Recently a special request for this information has been sent to the secretary of each society, and nearly all have responded, and corrections made accordingly. We hope that hereafter, advice of all changes will be sent to the editor of the REGISTER promptly.

